# Fidelity of kangaroo mother care services in the public health facilities in Bangladesh: a cross-sectional mixed-method study

**DOI:** 10.1186/s43058-021-00215-9

**Published:** 2021-10-09

**Authors:** Saima Mehjabeen, Mowtushi Matin, Rajat Das Gupta, Ipsita Sutradhar, Yameen Mazumder, Minjoon Kim, Shamina Sharmin, Jahurul Islam, Malabika Sarker

**Affiliations:** 1 Center of Excellence for Science of Implementation & Scale-Up (CoE-SISU), BRAC James P Grant School of Public Health, BRAC University, Dhaka, Bangladesh; 2 University of South Carolina, Columbia, USA; 3 Health Section, United Nations Children’s Fund (UNICEF), Dhaka, Bangladesh; 4 MNCAH, Directorate General of Health Services (DGHS), Dhaka, Bangladesh; 5 Heidelberg Institute of Global Health, Heidelberg University, Heidelberg, Germany

**Keywords:** Kangaroo mother care, KMC, Fidelity, Implementation research, Bangladesh

## Abstract

**Background:**

Kangaroo mother care (KMC) is a proven low-cost intervention to prevent neonatal mortality of pre-term and low birth weight babies and is very relevant to Bangladesh. KMC provides thermal regulation and thus directly avert neonatal mortality. KMC includes early, continuous, and prolonged skin-to-skin contact between an infant and caregiver, exclusive breastfeeding, early discharge from the hospital, and post-discharge follow-up. The purpose of this study was to investigate the fidelity of this intervention’s implementation according to national guidelines across all tiers of government (public) health facilities of Bangladesh.

**Methods:**

We adopted a triangulation mixed-methods approach of both quantitative and qualitative components in this research to support and explain the information obtained from quantitative observation with the help of qualitative interviews on the fidelity of KMC practice. We used an observation checklist to find the fidelity of KMC practice and used semi-structured guidelines to explain and understand the moderators of fidelity through key informant interviews and in-depth interviews. We undertook eight facility visits in four districts, observed twenty-three neonates and their caregivers during KMC practice at those facilities, and conducted twenty-seven key informant interviews with facility managers, health care providers, and five in-depth interviews with caregivers. Extracted information was triangulated and arranged under the themes of the fidelity framework.

**Results:**

Despite being a low-cost intervention, findings exhibit some adherence to the national guideline with several gaps in practice. Leadership played a critical role in ensuring the KMC practice. Specific components of KMC practice, like duration, nutrition maintenance, discharge criteria, and follow-up, were not consistent as recommended. Infrastructure, human resources, developmental partner support, and the demand-side and supply-side responsiveness played a critical role in enacting this human-centric approach’s fidelity. The observed interruption found in the implementation process posed threats to achieve the intended outcome as these caused violations of the basic principles of KMC.

**Conclusions:**

The study findings will help find ways to effectively deliver this intervention so that fidelity of practice is maintained, enhancing KMC services’ quality and advocating towards the successful scale-up of this program.

**Supplementary Information:**

The online version contains supplementary material available at 10.1186/s43058-021-00215-9.

Contributions to the literature/key findings

- Training of the health care providers with institutional support can facilitate the kangaroo mother care implementation.
- Contextualization of implementation strategies is mandated.
- Failure to adhere to the implementation plan and guideline may affect the achievement of the intended outcome.
- Community mobilization is crucial for the compliance and continuity of the intervention.
- Although cost-effective and less complex, implementing KMC in a resource-poor setting is yet challenging.

## Background

In recent years, health care systems have introduced many interventions, especially in low- and middle-income countries, to reduce neonatal mortality and morbidity and improve newborn outcomes. Kangaroo mother care (KMC) has proven to be a life-saving, low-cost intervention strategy to prevent neonatal mortality due to pre-term birth [1, 2]. KMC significantly decreases the risk of neonatal sepsis, hypoglycemia, and hypothermia, all of which contribute directly to neonatal mortality [3, 4]. It also aids optimal infant growth and development, parent-infant bonding, mother’s milk production, and parental emotional and psychological wellbeing [2, 3].

The World Health Organization (WHO) has officially endorsed KMC as a strategy to stabilize babies born with a birth weight of less than 2000 g in health facilities as a safe complement to conventional neonatal care [5–8]. Several studies pointed to the importance of KMC in pre-term, low birth weight babies and its potential for implementation at the community level and in other resource-limited settings [9–13]. Recent studies have identified several constraints towards the successful implementation of KMC in different health systems. Financial constraints and the poor recognition of KMC in national financial plans and policies, low budgetary allocation for the health facilities to scale up KMC, and the burden of out-of-pocket expenditures for caregivers are major constraints in this regard. Service delivery bottlenecks are physical and logistics constraints, lack of training, insufficient capacity development and mentorship, shortage of skilled health workforce, poor motivation, negative perception and attitude of health workers towards KMC due to inadequate knowledge, and weak health information systems [14–17]. Additionally, the clients’ challenges, such as existing socio-cultural barriers, the lack of acceptability of KMC, lack of engagement and support from fathers/male members in the community, and poor accessibility to the health centers make the successful implementation of KMC service challenging [7, 14–16]. These findings highlight the importance of in-depth examination of the implementation processes and a better understanding of the fidelity of a program to make it successful [12].

**Fig. 1:**
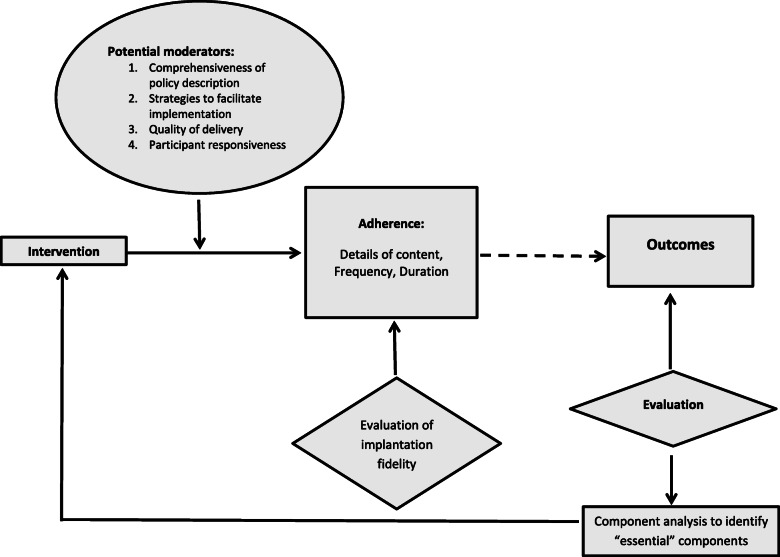
The framework of moderators influencing intervention-intended outcome

Implementation fidelity, or program integrity, refers to the degree to which programs or procedures are executed as planned [18–21]. Understanding the program’s fidelity is crucial because, despite the intervention’s efficacy, a public health program could not produce the desired impact if it fails to implement it with a certain degree of accuracy [13]. Carroll et al. introduced a modified conceptual framework of implementation fidelity, which outlines and explains the five classical fidelity elements’ functionalities: adherence, dose, quality of delivery, participant responsiveness, and program differentiation. These five elements, along with two additional features (i.e., intervention complexity and facilitation strategies), are crucial to achieving a higher fidelity level. Adherence, the underlining measurement of implementation fidelity, is defined as whether “the program, service or intervention is delivered as intended” [18]. The new conceptual framework of implementation fidelity aims to clarify further that the fidelity of an implementation approach cannot be captured independently from adaptation as both are intrinsically linked [22]. Dosage (dose delivered) and exposure (dose received) refers to “whether the frequency and duration of the intervention are as complete as prescribed” [13, 18]. Coverage is defined as “whether all the people who should be participating in or receiving the benefits of an intervention, in reality, do so” [19, 20]. Moderators, i.e., factors that may influence the degree of fidelity, must be considered when evaluating the relationship between interventions and their intended outcomes, as shown in Fig. 1. The introduction of facilitation strategies and intervention complexity aims to advance understanding of the degree of implementation fidelity has on the intended outcomes. Suitable facilitation strategies improve the chance for greater organized fidelity in a program or service [19, 20]. The comprehensiveness of policy or program description of an intervention is more likely to be enforced with high fidelity than a generic description [19, 20].

To end preventable child deaths by 2035, the Ministry of Health and Family Welfare, Government of Bangladesh has declared KMC a critical intervention and recommended nationwide scale-up. In 2015, the National Technical Working Committee (NTWC) on Newborn Health formulated the KMC National Guideline (Annex 1) and Training Manual on KMC for Bangladesh to facilitate the process [10]. Several studies also showed that KMC service could be successfully implemented in community and resource-limited hospitals in Bangladesh [10–12]. Despite this, only a few health facilities have introduced KMC services in Bangladesh [7, 8]. So far, no study has been conducted in this country to understand the implementation fidelity of KMC. Therefore, we conducted this study to assess the implementation fidelity of KMC service in Bangladesh’s selected health facilities. This study also aimed to explore the facilitators and challenges of implementation of KMC service at different levels of health facilities in Bangladesh. We expect the findings of this study would complement the scale-up strategy of KMC programs in Bangladesh.

## Methods

### Study approach and study design

We adopted a concurrent triangulation mixed-methods approach using both quantitative and qualitative methods. The purpose of this design is “to obtain different but complementary data on the same topic” [23]. The quantitative component intended to identify the fidelity of KMC practice, and the qualitative component aimed to understand the facilitators and challenges of implementing KMC service.

### Study duration and study site

The study was conducted between April 2018 and October 2018. The study sites were eight purposively selected health facilities in four districts across Bangladesh (Dhaka: Dhaka Medical College Hospital and Institute of Child and Mother Health; Kushtia: Kushtia District Hospital and Kumarkhali Upazilla Health Complex; Kurigram: Kurigram District Hospital and Ulipur Upazilla Health Complex; Tangail: Tangail District Hospital and Kalihati Upazilla Health Complex). We selected these health facilities because KMC service was either implemented (six health facilities) or planned to be implemented (two health facilities) in these health facilities. The study sites were primary level (Upazilla Health Complex), secondary level (District Hospital), and tertiary level health care facilities (Medical college hospital/specialized hospital). It was likely that the implementation fidelity, as well as the facilitators and challenges related to the KMC implementation, would vary at different levels. In two facilities, the planning process to roll out KMC services was in progress during data collection. We limited our study to the government health facilities as the government of Bangladesh had a plan to scale up KMC service across the country.

### Study population and sampling technique

Study populations were health providers (doctors and nurses) and health facility managers (Upazilla Health & Family Planning Officers, Superintendents, and Hospital Directors who were the highest administrative authority of Upazilla Health Complexes, District Hospitals, and Specialized Hospitals). We also interviewed mothers and caregivers of KMC babies. Health providers and health facility managers were selected purposively based on their involvement in the KMC implementation process at the selected health facilities. The mothers and caregivers were selected conveniently based on their availability at selected health facilities during the data collection period.

### Study tool

The research team developed the context-specific tools. The observation checklist (Annex 2) used for the quantitative component was prepared based on the “National Guideline for KMC in Bangladesh.” The semi-structured guidelines applied to the qualitative component followed the “WHO health system building blocks framework” (Annex 3). Before initiation of data collection, all tools were pre-tested at different health facilities providing KMC to clarify the instruments, redundancy, comprehensiveness, and length of the time to be used and revised accordingly.

### Data collection process

Four research teams, each comprised one senior researcher, one medical doctor, three junior researchers, and one field coordinator, were involved in data collection. Senior researchers conducted a 2-day training session for the junior researchers and field coordinators prior to the data collection.

### Quantitative component

To gather information on KMC implementation fidelity, we observed the infrastructure for KMC provision (e.g., availability of KMC room, weight machine, health education material, record-keeping system) in all eight health facilities. We also observed mothers and caregivers of 23 neonates while delivering KMC to their babies in five health facilities. Observation of KMC practice was unreasoned for the rest of the three health facilities because either KMC provision was temporarily ceased due to renovation activity or KMC service had not been started during our data collection period. In each health facility, the observation was carried out for two consecutive weeks. The reason for observing the KMC sessions for 2 weeks was to avoid the Hawthorne effect [22]. During the data collection period, research teams carried out observation every day from 9:00 am to 3:00 pm.

### Qualitative components

We conducted 27 KIIs with the health providers and facility managers and five IDIs with mothers or caregivers of KMC babies in the selected health facilities until data saturation had been achieved [23]. All interviews were held in the respondents’ preferred places at respective health facilities to ensure adequate privacy. Interviews were recorded using audio recorders, and field notes were taken. Informed written consent was obtained from each participant.

### Data analysis

#### Quantitative component

Upon completing data collection, quantitative data were entered in Microsoft Excel and converted into a STATA file format. The data manager cleaned the data after checking the variable names and labels. The recording of values and categorization of variables was done to facilitate further analysis. Descriptive analysis, such as frequency estimation, was done to find out the fidelity of KMC practice. The statistical analysis was performed in STATA version 13.

For the qualitative component, interviews were transcribed verbatim into Bengali and translated into English. Transcriptions were read repeatedly to achieve data familiarization and coded using both deductive and inductive approaches. For deductive coding, an a priori codebook was developed following the fidelity framework suggested by Carroll et al. [18]. The members of the research team checked data to ensure intercoder reliability. Subsequently, a data display matrix was developed, including appropriate quotes to summarize the findings. Finally, thematic analysis was performed to describe the facilitators and challenges of KMC implementation.

#### Triangulation

During data analysis, triangulation of the quantitative and qualitative data was performed to compare the findings obtained from observation of KMC practice with the information collected through the interview with health providers, managers, and caregivers.

## Results

### Implementation fidelity

#### KMC practice

The average birth weight of the observed babies was 1568 g, and the range was between 1000 g and 2100 g. In all the health facilities, newly admitted/delivered and unstable LBW and/or preterm babies were referred to the SCANU and referred to the KMC room after stabilization. In contrast, stable babies were directly sent to the KMC rooms. All the health facilities were found to provide either intermittent or continuous KMC service. Only two health facilities allowed mothers/caregivers to offer KMC service for 24 h per day. Most mothers/caregivers were discharged from the hospital on request before their babies had gained adequate weight recommended by doctors. Post-discharge follow-up was advised in most of the cases. The summary of the study has been presented in Table 1, followed by a detailed description.

**Table 1:** Kangaroo Mother Care (KMC) implementation fidelity in selected health facilities in Bangladesh

Health facilities		Eight; KMC babies observed in five facilities
**Respondents**	Demand-side	• 23 neonates observed • 19 KMC providers
	Supply-side	• 27 KIIs • Five IDIs
**Themes according to CFIF**		**Main findings**
**Coverage**		78%; 18 neonates
**Content**		Counseling—not regular, coms material use—65% 15 neonates, clothing of the baby—not followed completely
		Quality of delivery—none of the health facilities maintained all KMC criteria
		Position of KMC—eighteen (78.2%) babies maintained KMC position
		Sleeping and resting position of mother—twenty (87%) mothers/caregivers maintained the position
		Feeding—according to the record, nutrition maintenance was observed in 13 neonates (56%)
		Clinical examination—not done six-hourly or regularly
		Discharge criteria—only one neonate met all discharge criteria
		Discharge criteria for mother/caregivers—only one mother met all discharge criteria
		Routine follow-up—not well maintained
		Record-keeping and documentation available on KMC practice—none of the facilities (0/5) maintained all criteria for record-keeping of KMC practice
**Dose**		No neonate received 20+ hours KMC per day
**Moderators**	Intervention complexity	Many components need staff training, strict supervision, continuous clinical monitoring, and follow-up
	Challenges in providing quality of care	• Shortage of workforce and heavy workload • Lack of motivation among service providers • Role of caregivers • The unwillingness of caregivers to stay at the hospital • Financial constraints • Hot and humid weather • Post-discharge follow-up
	Facilitation strategies	• The policy and stakeholder support • Leadership & KMC champions
**Participant responsiveness**		Both service providers and caregivers shared that KMC is an effective intervention for pre-term and low birth weight management. In most cases, KMC uptake was good, and both service providers and caregivers were engaged.

#### Coverage

In this study, 18 out of 23 (78%) neonates met all the inclusion criteria for receiving KMC (being LBW; the willingness along with full-time availability and support for mothers or immediate family members for starting KMC).

#### Content

##### Infrastructure

A dedicated room for KMC provision is essential for the proper implementation of the service. All five health facilities had separate rooms where mothers/caregivers used to provide KMC to their babies. In most cases, mothers/caregivers had to share rooms with other caregivers. Three facilities had curtains though maintaining privacy was challenging as outsiders could enter into KMC rooms at any time. In secondary and tertiary level health facilities, the KMC rooms had air conditioning facilities; however, those were functional only in tertiary hospitals. KMC rooms in all health facilities had fans, lights, and televisions. Adjustable beds for mothers/caregivers were available in few facilities, whereas specially designed pillows were used to make the beds comfortable in other sites. Though the majority of the health facilities had proper handwashing stalls (5) and toilet facilities with running water (4), mothers/caregivers rarely maintained personal hygiene. The availability and usage of hand sanitizer by health providers were also infrequent. None of the health facilities had all types of equipment required to provide KMC maintaining the highest standard.

##### Positioning of babies and mothers/caregivers

Among 23 babies observed, 18 (78.2%) received KMC maintaining proper position (placed in the upright position keeping baby’s abdomen at the level of and in between mother’s chest with the support of a binder from the bottom, turned baby’s head to one side, hips slightly extended in a frog-like position). Besides, in 20 cases (87%), mothers/caregivers maintained the recommended reclined/semi-recumbent position while sleeping or taking rest.

##### Clothing and feeding practice of babies

We found that 18 out of 23 babies (78.2%) were wrapped with diapers during observation. Sleeveless shirts and caps were used as diapers for five babies. We also found three babies with their socks on. Exclusive breastfeeding was ensured for 12 babies, and alternative feeding methods like nasogastric feeding and feeding expressed breast milk using a spoon was practiced for eight and six babies, respectively. Only 13 (56%) mothers/caregivers followed the breast/alternative feeding guideline available in the KMC rooms of three health facilities.

##### Educating mothers/caregivers

Health education materials are an essential component of KMC service provision as beneficiaries can be exposed to those and learn about the intervention. All five health facilities supplied health education materials like brochures and leaflets to the mothers/caregivers. In these materials, different aspects of KMC service (e.g., importance and benefits of KMC, methods of providing KMC, maintaining proper position for babies, proper use of binders) were portrayed using easily understandable words and pictures. In four health facilities, KMC-related posters displayed the success stories. In three facilities, KMC rooms had televisions that showed videos on KMC service to mothers/caregivers.

##### Counseling mothers/caregivers

Appropriate counseling during admission and discharge of KMC babies is an integral part of the successful implementation of KMC service. In the health facilities we studied, health providers occasionally counseled admitted mothers, caregivers, and family members to motivate them to continue KMC service. Health providers also counseled mothers/caregivers during discharge, mainly on the methods of providing KMC at home and the importance of regular follow-up, but the mothers who left the hospital by discharge on risk bond (DORB) did not receive any counseling. Nevertheless, in most cases, counseling service was neither comprehensive nor provided regularly. For instance, during observation, we found that before initiation of KMC service, nurses briefly demonstrated the steps and duration of providing KMC service but skipped the importance and benefits of KMC that could have been motivated mothers to continue the care. In one health facility, nurses were not responsive while counseling mothers/caregivers.

##### Clinical examination

Doctors and nurses observed the breathing, heart rate, and wellbeing of all 23 neonates (100%) at least once daily. Only ten neonates’ daily weight and seven neonates’ six-hourly temperature were checked for three consecutive days, and weekly head circumference was measured for only one of them. The routine weighing was not done regularly for any neonate.

##### Discharge criteria for babies and mothers

Only one neonate and one mother met all discharge criteria. Among 23 neonates, four were shifted to the special care neonatal unit (SCANU), four left with DORB, and four were sent to the neonatal intensive care (NICU). Caregivers of three KMC babies left the hospital without any notice, and five were not discharged until the last day of data collection. Health providers checked all nine discharged neonates for nutrition criteria (on breastfed or cup/spoon-fed). Target weight gain, temperature maintenance, and oxygen requirement were checked for three consecutive days for four neonates.

##### Routine follow-up

Only one mother who participated in this study returned to the health facility for follow-up during the data collection period.

##### Availability of record-keeping documents and guideline

Though the KMC Register and KMC Record Form (to be filled in by a doctor) were present in all facilities, none of the facilities maintained all criteria for record-keeping of KMC practice. However, a daily follow-up chart for the neonates (to be filled in by a nurse), a KMC monthly progress report, and follow-up cards were found in four facilities. A copy of the KMC National Guideline was found in two tertiary facilities.

#### Dose

##### Duration of KMC

The outcome of KMC service depends on the duration of service provision to a great extent. According to the daily follow-up reports, only 15 (65.2%) received continuous KMC. Amid the rest, six and two babies received intermittent and sporadic KMC service, respectively. For 18 (78.2%) neonates, the duration and timing of KMC provision for the previous 24 h were recorded daily.

### Moderators

#### Intervention complexity

The KMC intervention is one of the simplest interventions in the public health field. National KMC guidelines and BCC materials are detailed and straightforward, depicting every step specifically, but these were not available in most facilities. The detailed and clear recommendations of BCC materials with explanatory illustrations were beneficial (if available) for the mothers/caregivers for easy understanding. Keeping the babies for a long time in the kangaroo position, feeding them in that position, and following up after discharge were complex components and had some variations in their delivery with inadequate record keeping.

### Challenges of providing KMC

#### Shortage of trained workforce and heavy workload

Almost all health providers and facility managers who participated in this study stated that KMC service maintaining the highest quality at their health facilities was disrupted due to a shortage of trained workforce. For instance, health providers of a tertiary hospital stated that they could not provide KMC during night time (8 pm–9 am) because of a lack of adequate workforce. As a result, they shift the babies from the KMC room to SCABU after 8 pm. In this regard, one participant said,

If six doctors do the work of twenty-seven doctors, it is quite impossible to maintain the quality of the work. (KII 4_Primary2_ Upazila Health &Family Planning Officer)

Due to a workforce shortage, health providers need to work in different hospital units, which poses a heavy workload. As a result, they cannot offer adequate time and attention to provide KMC service.

Three of us are in the duty, and all of three are present, but still we have a lot of patients, there are delivery cases, other cases and also the follow up of the KMC patient. We come periodically but cannot stay continuously with the mother. (KII 2_Primary 1_Senior Staff Nurse)

#### Lack of motivation among mothers and service providers

According to several respondents, mothers/caregivers often become demotivated to continue KMC, as the effect of the KMC cannot be experienced immediately. A few health providers also perceived KMC service provision as an additional burden and tried to avoid it by not admitting eligible neonates. In one respondent’s voice,

As far as I know, sometimes consultant physicians are not oriented and cooperative and make negative remarks about KMC. They are very knowledgeable, and they do not need training, but they need motivation. (KII 4_Primary 2_Upazila Health & Family Planning Officer)

According to few providers, another demotivating factor was the absence of financial remuneration for health workers.

#### Lack of engagement with mothers

Communication with the KMC caregiver, primarily the mother, is crucial for regular KMC practice. However, mothers were not adequately engaged in the decision-making process and counseling sessions in our study sites. Instead, health providers preferred to conduct the sessions with their husbands or mothers-in-law.

#### The unwillingness of caregivers to stay at the hospital

Several study participants reported that KMC mothers were reluctant to stay in the hospital for an extended period. The reasons they mentioned were a sense of guilt for not performing the responsibility of household chores for a long time, inability to take care of their other children at home, and lack of support from husbands, mothers-in-law, and sisters-in-law.

We do not have any complaints about the hospital. We are satisfied with the services they provide. However, it is my problem. I cannot stay here longer. I have many responsibilities in my household. I know it would be better for the baby and the mother to stay here for a few more days. But what can I do? (IDI 3_Primary 1_Caregiver)

#### Lack of supportive family members

Mothers sometimes sought early discharge because of the unwillingness of their family members to stay with them at the hospital. All respondents (service providers and mothers) agreed that it is quite impossible for KMC mothers to stay at the hospital for a long time without family members’ support. However, in most of the facilities, no arrangement (e.g., beds for sleeping, chairs, separate toilet facility) was available for the attendants. Moreover, longer hospital stays disrupted earning of the male family members (husband, father, brother) and interfered in performing the daily household chores of female family members (sister, mother, sister-in-law, and mother-in-law). Attendants also needed to buy food and pay for the transportation from and to the hospital, which posed a financial burden to the family. In this regard, one health provider said,

Most of the patients we receive here are from low socioeconomic status. They usually do not show enthusiasm to keep babies and mothers in the hospital for a long time. Men do this because most of them are day laborers, and if they cannot go for work for a single day, they might remain hungry for the next day. (KII 2_Primary 1_Senior Staff Nurse)

#### Out of pocket expenditure

There is no annual budget specific for KMC service in primary-level hospitals, and KMC logistics (like diapers) shortages were common. The caregivers needed to buy diapers from outside, an additional cost for the low-income families. These challenges often impede successful KMC implementation. On respondent, in this regard, stated,

No, we do not have a supply of diapers from the hospital. Many poor mothers cannot buy diapers.it would be very good if adequate logistic supply is there, including diapers for babies, dresses, and better-quality food for the mothers. (KII 2_Primary 1_Senior Staff Nurse)

#### Hot and humid weather

The crucial challenge of providing continuous KMC in primary level hospitals was hot and humid weather. Though tertiary hospitals had functioning air conditioners at the KMC rooms, this facility was unavailable in primary and secondary hospitals. Therefore, mothers continue to sweat during skin-to-skin contact practice, which was very uncomfortable. Thus, mothers and other caregivers were reluctant to continue KMC for prolonged hours. One facility manager said,

Though we have 24/7 electricity supply in our hospital, mothers suffer a lot while providing KMC, especially during summer. We do not have a facility for air conditioning. It is not possible though to use the air conditioner at KMC rooms because it may cause hypothermia of patients. (KII 4_Primary 2_Upazila Health & Family Planning Officer)

#### Reluctance to attend health facility for post-discharge follow-up

Adherence to post-discharge follow-up was also challenging due to a poor understanding of the KMC benefits. Long-distance transportation expense necessary for follow-up visits was also a common barrier. Health providers contacted the family over the phone and tried to motivate them to visit the health facilities. Sometimes, community health workers visit the child’s house to counsel parents to come to the hospital for follow-up. One of our respondents said,

In many cases, patient parties willingly come here for a follow-up though some parents do not come. In that case, we contact them over the phone. In fact, we note their phone number during discharge. However, if it does not work, our assigned Health Assistants visit the baby’s house and request them to bring their baby to the hospital for follow-up. (KII 4_ Secondary 2_Senior Staff Nurse)

### Infrastructure

#### Inadequate logistic supply

Health providers and facility managers often stated that inadequate logistic supply (e.g., binder, diaper, caps, and socks) disrupts the KMC service provision at their facilities. They tried to generate an alternative source of funding; otherwise, they requested the families to purchase. One facility manager said,

For some products (binder and initial furniture like bed, pillow, chair, KMC room), there was enough funding from Save the Children. However, when logistic supply was stopped, we started to manage the products from government supply. We were lucky that we received enormous support from the local government. Occasionally local government allocates several funds that can be used in the health sector. Representatives of local government (UP Chairmen, Members, Upazila Nirbahi Officer/UNO) allowed us to use this fund to purchase KMC related products. (KII 4_Primary 2_Upazila Health & Family Planning Officer)

He added,

In case of some logistics such as a diaper, there is no supply, and we also cannot arrange this. We usually ask the patient party to purchase this. However, when I go to any training, meeting, or forum, I raise this issue, and I hope we will receive this from the government soon. If it happens, it will hasten the success of this intervention. (KII 4_Primary 2_Upazila Health & Family Planning Officer)

#### Unavailability of separate toilets for KMC mothers

There was no separate toilet facility for KMC mothers in several health facilities, and mothers were discouraged from staying at the facility and continuing KMC for the recommended period. One respondent said,

The mothers have to suffer a bit to go to the toilet, which is far away from the KMC room. This is a problem. If there was a toilet was adjacent to KMC room, it would be great. (KII 4_ Secondary 2_Senior Staff Nurse)

#### Unavailability of the adjustable bed

Unavailability of adjustable beds sometimes made KMC service provision challenging for mothers/caregivers. The challenge was sometimes solved by using pillows; however, it could not provide proper comfort to KMC mothers while providing care. One provider said,

We do not have an adjustable bed. Here, we try to make the beds comfortable using a pillow. But it would have been better if we had the beds raised at a 45° angle. The mother would have felt better. (KII 2_DMCH_MO)

### Facilitators for providing KMC service

#### Leadership and KMC champions

Dynamic leadership, personal experience, and previous exposure to the KMC service of the facility managers and service providers in four facilities created an enabling environment. In their opinion, KMC is an effortless, sustainable life-saving procedure. Self-motivation and the extraordinary commitment of providers played a significant role in facilitating the KMC program. Service providers were happy with the positive outcome of the KMC practice. They were further encouraged to continue the KMC service after witnessing the baby’s improved condition in the facility and post-discharge follow-up. Watching caregivers provide KMC and understand the KMC concept and its importance was also motivating.

One advantage of KMC is that if a baby is suffering from hypothermia, we can save the baby therefore not requiring them to go far for additional health services. This is beneficial to them. (KII 4_ Primary 3_Senior Staff Nurse)

The local resource was used for making KMC binders, and extra support was provided by local clothing pieces like an “orna” (lady’s scarf) to keep the babies in the KMC position. Multiple pillows were used if the beds were non-adjustable or if a special KMC support pillow was absent. Repeated wiping of the neonate and mother for excessive sweating was done to combat the heat and humidity-related problems.

In one tertiary facility and one primary facility, three meals per day were offered. At the same time, the hospital authority of another primary facility provided additional accommodation for one to two family members. In some facilities, health providers arranged food for the attendants to encourage the patient’s attendant/caregiver to stay at the hospital in the empty patients’ beds. The facility manager said,

Sometimes we try to provide meals to attendants who stay with KMC mothers. However, it is not possible always. (KII 3_Primary_1_UH&FPO)

#### Training of the health providers

In many facilities, trained health providers coupled with structured monitoring and supervision enabled successful KMC service implementation. However, the health providers’ training varied across the health facilities and provided training wings of different organizations. Although many revealed that training improved the knowledge and skill for implementing KMC, there were no provisions to run refresher training for doctors and nurses in any facilities.

#### Monitoring

The Quality Improvement Initiative (QII) program, with a development partner and GOB support, was responsible for monitoring the KMC service in one health facility. A designated nurse maintained the register book and updated the follow-up routine information every two hourly. The medical officer or consultant checked the information while visiting patients. Regular quarterly visits and measuring performance scores performed by development partners were a driving force for better performance in providing KMC services. In another facility, two doctors from another project, nurses, the residential medical officer, and a UHFPO monitored KMC. They used a checklist for monitoring by cross-checking and using the standard operating procedure. Monthly meetings were held in one tertiary care facility to address the challenges and discuss KMC service improvements. Periodic follow-up and record-keeping as part of monitoring and supervision ensure the quality of the KMC service provision. However, regular follow-up data was incomplete in some cases. Statisticians at the facilities were responsible for sending monthly reports to the Civil Surgeon Office and central HMIS.

#### Participant responsiveness

Both service providers and caregivers shared that KMC is an effective intervention for pre-term and low birth weight management. In most cases, KMC uptake was satisfactory, and both service providers and caregivers were engaged. Families accepted the KMC when they understood the process and its role in the child’s wellbeing.

The majority of the facility managers and service providers were enthusiastic and committed to implementing KMC even with continuous challenges. Occasionally, service providers were demotivated because of the high workload, inadequate training and support, and human resource shortage.

#### Mothers’ education

The mother’s education played a critical role in the successful implementation of the KMC program. Educated mothers could grasp the KMC-related health education materials better and realize the importance of KMC and practice it.

It is easy to make educated mothers understand the benefits of KMC. They follow our instructions without a doubt… Educated mothers also can read KMC related manuals. (KII 2_Primary 1_Senior Staff Nurse)

## Discussion

It is the first study that assessed the fidelity of KMC in public health facilities in Bangladesh. Most health facilities adhered to the national KMC guideline, but inconsistencies between the guideline and practice varied noticeably [24]. Context influences the implementation process, and occasional discrepancies in implementing the interventions are inevitable [25, 26]. Most challenges were related to continuity of care and post-discharge follow-up, as previously observed in other low-income country contexts [27, 28]. Strong leadership and “champions” facilitated the intervention and paved the way to the sustained practice of KMC. Studies conducted in other Asian and African countries have reported similar findings [28–31].

The study revealed that full adherence to KMC practice as per the guidelines was impossible in many observed cases due to the inadequate infrastructure, shortage of experienced workforce, and supportive environment. Many health facilities made sincere attempts to follow the national guideline, especially in selecting eligible KMC candidates. The majority of the KMC babies did not meet the discharge criteria, and there was no proper follow-up once the mother was discharged from the health facility. Community participation and buy-in for follow-up were also absent. All facilities maintained a record-keeping system to some extent with scope for further improvement.

The quality of implementation influences its effectiveness and outcome as in other interventions [26]. The observed gaps in implementation are quite alarming because these steps form the core foundation of any KMC practice. Such deviations can undermine the intervention’s ability to achieve its intended purpose [13]. Temperature maintenance without conventional incubator care in NICU, SCANU, and decreasing the risk of neonatal sepsis, hypoglycemia, and hypothermia, promoting weight gain and fewer hospital-acquired infections are essential for ensuring quality KMC and have been proven elsewhere [4, 32, 33]. If the desired outcome of KMC is not achieved, neither the service providers nor the caregivers will be interested in implementing and following the KMC strategy according to the standard. They will prefer to place the newborn in incubators rather than make additional efforts for carrying out KMC, the most cost-effective and human-centric care model. Therefore, this will only result in an extended hospital stay, raise expenses, and increase chances of adverse outcomes like sepsis. In KMC, mere skin-to-skin contact is not sufficient. The proper positioning of the baby, adequate duration of KMC, maintaining nutrition, and ensuring follow-up are equally, if not more, critical for the successful implementation of KMC. A recent systematic review and meta-analysis show that disrupted duration of the KMC has a negative impact on a child’s growth [34]. The caregiver needs to understand and accept it and seek help when needed. Countries with complete adherence to the KMC guideline result in health benefits for LBW infants; ensured better survival outcomes, increased growth parameters, shortened duration of hospitalization, and reduced cost [32].

It is apparent that demotivated health care providers’ and caregivers” reluctance seriously impedes the implementation of KMC in Bangladesh, as observed in other low-income countries, namely Vietnam, Malawi, Uganda, and Mali [14, 17, 30, 35], which affected the fidelity of KMC. Across all health facilities visited, the prevailing health system challenges were inadequate and skilled human resources and excessive workload. The lack of qualified workers to provide quality patient service failed to adhere to the KMC guideline [27]. Moreover, a wastage of the trained workforce due to the rotation in different departments, turnover, and lack of staff orientation is common [7, 15–17, 29]. Implementing facilities should consider a panel of qualified and inspired health care professionals in designing any implementation strategy [36] for an intervention like KMC [4, 14]. Selection of staff with adequate training, belief in intervention efficacy, and positive attitude coupled with enabling institutional policies will result in high implementation fidelity, as evidenced by other settings [7, 16, 37]. Existing literature recommends managerial support, effective communication, and limited (or zero) staff rotation as positive determinants for KMC [17, 27].

Well-trained and motivated health workers can provide quality counseling to mothers and family members, especially mothers-in-law and husbands, for ensuring fidelity and service quality. BCC materials are instrumental for information clarity for both the service providers and caregivers [29–31, 38]. Implementers have to emphasize the correct use of BCC materials.

Evidence suggests that KMC should be started intermittently from NICU/SCANU and continued after shifting to the KMC room under the continuous supervision of doctors and nurses [17, 39]. This supervised initiation will result in better management and confidence building of the care provider. A separate space for KMC, located near the maternity ward and connected with neonatal care, can facilitate early initiation [4, 17, 29–31].

Continuity of KMC practice following initiation and early discharge from the health facility is vital for improved newborn outcomes. Early discharge is a crucial feature and core component of KMC and a prerequisite for the national KMC guideline [24]. Unfortunately, a systematic post-discharge follow-up strategy for facility and community was absent similar to other countries like India, Indonesia, and the Philippines [28, 29]. An effective follow-up includes formal preparation and adequate counseling of the mother/family member and community awareness [28]. Sensitization during antenatal care and community health worker empowerment can be feasible solutions in this regard [27, 28, 39]. The literature recommends developing a strong referral link between health facilities and community-based follow-up of neonates with low birth weight (LBW). CHWs’ role in encouraging caregivers for a follow-up visit and KMC follow-up as components of existing postnatal services is essential [17]. A partnership between the government and NGO with the integration of community-based care might improve this condition. Furthermore, a robust HMIS with the integration of KMC services is required to ensure monitoring and evaluation [29].

According to the guideline, if health facilities do not implement KMC, it is likely to raise questions about the intervention’s usefulness in Bangladesh. A contextual adaptation with relative compliance while retaining core elements ensures the intervention’s effectiveness and viability [26, 40]. The physical presence of a written policy and national guidelines and its adaptation and adherence at the specific level of health care facilities will aid in implementing KMC services [28]. The investment in infrastructure for baby and mother and adequate training of the health providers are crucial for a successful KMC program, as suggested in the international KMC conference [41].

### Strengths and limitations

One of the significant strengths of this study was its rigorous methodology. Additionally, we collected data from health providers, administrators, and caregivers of KMC babies, ensuring a comprehensive understanding of KMC implementation in the selected health facilities. Our study considered content and process fidelity as a whole and observed whether front-line workers implemented them according to the national guideline [26]. This approach offered a comprehensive picture of implementation fidelity and would be insightful for planning scale-up. However, this study is not free from limitations. The first limitation emerged during data collection, as we could only observe 23 babies, and the second one is the failure to include three facilities that were not offering KMC practice. Due to resource constraints, we excluded private health facilities (for observation) and interviewing other attendants presented at the health facilities (e.g., fathers, aunts, grandmothers).

## Conclusion

The concept of KMC is relatively new in Bangladesh. We are hopeful that the policymakers and key implementers will use the study findings to improve KMC services’ quality and the scale-up of the program in Bangladesh. Despite many challenges, leadership and commitment exemplified by facility managers and a team of dedicated and trained health care providers played a significant role in facilitating KMC services. The absence of proper counseling, lack of a structured post-discharge follow-up, and poor continuity-of-care from facility to community level are noted among the many difficulties observed. Poor implementation of the core activities of KMC delays the intended outcomes and could result in program failure if left unaddressed. There remains a lot of scope and challenges to adapt the national guideline with the implementation context at different levels. The effective interpersonal counseling of the service seekers by trained health staff using appropriate BCC materials, patient-friendly support to caregivers, and the family members (key decision-makers) to support KMC will go a long way in improving the fidelity of the interventions.

## Supplementary Information

**Additional file 1.**

## Data Availability

The BRAC JPGSPH does not yet have any data repository system. All transcripts and the facility data are saved in a folder under Institutional Review Board. The IRB chair is Professor Dr. Syed Masud Ahmed (email: ahmed.sm@bracu.ac.bd), and the co-chair is Associate Scientist Dr. Atonu Rabbani (atonu.rabbani@barcu.ac.bd). They will be able to provide the transcripts and facility checklist data.
