# Supplementary material for: Fidelity of kangaroo mother care services in the public health facilities in Bangladesh: a cross-sectional mixed-method study
Source: Implement Sci Commun. 2021 Oct 9;2:115. doi: 10.1186/s43058-021-00215-9 (PMC8501568; doi:10.1186/s43058-021-00215-9)
Supplement: Supplementary file 1 — Additional file 1. [file 43058_2021_215_MOESM1_ESM.docx]

**Annex 1**

**Kangaroo Mother Care according to the National Guideline, Bangladesh**

KMC is skin-to-skin contact between the newborn and the mother or the caregiver for pre-term and/or LBW newborn**.** The newborn is kept in the upright position in between the mother's breast, head turned to one side, slightly extended hips in a frog-like position, the baby's abdomen at the level of the mother's chest, and support from the bottom with a binder. It includes:

Early, continuous, and prolonged skin-to-skin contact between the
mother/caregivers and the newborn;

Exclusive breastfeeding

Early discharge

Continuation at home

Adequate support for mothers and family at facility and home;

Follow-up at home [National Guideline on Kangaroo Mother Care (KMC) Page 9- 10]

**Inclusion criteria:** The baby birth weight should be <2000 grams to initiate KMC for a stable pre-term baby and/or low birth weight newborn. The other criteria are the mother/caregiver's willingness to provide KMC, full-time availability, and family members' support.

**Counseling:** Using appropriate communication materials coupled with extensive interaction with the mother and other family members (i.e., mother/mother-in-law, husband, etc.) to help create a supportive environment are prerequisites for KMC.

**Information about KMC**: KMC related information in the form of Behavior Change Material (BCC) materials helps motivate the mother/caregiver. Such as brochures and information sheets, KMC posters on display, and videos.

**KMC logistics and supplies:** Beds with adequate back support and pillows, comfortable armchairs, binders to hold the infant in the KMC position, a curtain for privacy, diapers, a fan or air conditioning unit, a TV for health information/recreation, and a nasogastric tube are necessary to provide KMC. A hand washing provision, a toilet facility with water, waiting for space for KMC providers (mother or any family member), and the maintenance of optimum room temperature.

**Clothing of the baby:** A baby should wear a cap, socks, a nappy, and a sleeveless shirt open in the front.

**Duration of KMC:** KMC should start in the facility/ward immediately after birth in a stable eligible baby. KMC should be practiced continuously for more than 20 hours per day. Sessions with fewer than two hours should be avoided because frequent handling may be stressful for the newborn.

**Position of KMC:** The baby must be placed upright in between the mother's breasts, with the head turned to one side, and slightly extended; hips e in a 'frog-like position, with the baby's abdomen at the mother's chest level and a binder to support the baby from the bottom.

**Sleeping and resting position of KMC provider:** A reclined or semi-recumbent position, about 30-40 degrees from horizontal, for comfort during sleep. The resting position can be ensured with an adjustable bed or with several pillows on a non-adjustable bed.

**Feeding:** The mother may express her breast milk while the newborn is still in the KMC position. The newborn can be fed with a cup, spoon, or feeding tube, depending on the newborn's condition. The mother must clean her hands with soap and water before each feeding. The feeding frequency will depend on the quantity of milk the newborn can tolerate per feed and the required daily amount, and the amount per feed for small and pre-term newborns should be steadily increased.

**Clinical examination:** The baby's temperature must be measured every six hours per day for three consecutive days. The information about weight gain**,** breathing patterns, heart rate, oxygen intake, weekly head circumference measurements should be collected and recorded regularly.

**Discharge criteria:** For three consecutive days- the baby must be fed by breast or cup/spoon-fed and gain approximately 10-20 gm/kg per day; the temperature must be within the normal range while in the KMC position (axillary temperature of 97.6℉ - 99.4℉), the baby should not require additional treatment for oxygen and the baby's condition must be stable with no signs of infection, illness, or other danger signs.

**Discharge criteria for KMC provider:** The KMC provider must be confident in KMC practices and breastfeed or cup/spoon-feed the baby. The service provider must explain to the mother how to properly perform KMC practices at home, and the mother must be able to recognize the danger signs and when to return to the facility for help.

**Routine follow-up**: Routine follow-up of the baby will be done at the household by the Community Health Workers (CHWs). If necessary, the mother should visit the facility for follow-up.

**Record-keeping/availability of documentation on KMC practice:** There are four different essential documents or forms related to KMC: (1) KMC National Guideline, (2) KMC register, (3) KMC booklet, and (4) follow up cards. KMC record forms are to be filled in by the doctors, and the daily follow-up charts for the baby are to be filled in by the nurse. The health provider in charge of the KMC unit is responsible for filling in KMC services monthly progress reports.

For this study, we used the following definitions for three types of KMC practice-

**Continuous KMC:** Baby is in the KMC position >20 hours + per day

**Intermittent KMC:** Baby is in KMC position at least once or twice per day for at

least 60 minutes

**Sporadic KMC:** Baby is in KMC position for less than 60 minutes per day and not done every day.

**Annex 2**

**Table 2: Observation findings on KMC practice in the selected facilities**

| **Variables** | **Total Number of Babies Observed (n=23)** |
| --- | --- |
| **1. Weight** |  |
| - Weighed at birth | 22/23 |
| - The baby was underweight for initiating KMC | 22/23 |
| - Functioning weighing machine present in the ward **(n=5 facilities)** | 5/5 |
| **2. Inclusion Criteria** |  |
| - Weight of the baby was ≤2000 gram | 22/23 |
| - Mother /KMC provider willing to provide KMC | 22/23 |
| - Mother /KMC provider fulltime available | 20/23 |
| - Other family members available to support mother/KMC provider | 20/23 |
| **Presence of all domains** | 18/23 |
| **3. Counselling:** |  |
| - Mother/Father/Family Member counselled for KMC | 19/23 |
| - Presence& use of, Behavior Change Communication material, KMC flip chart &Counselling board during counselling (n=23) | 15/23 |
| **Presence of all domains** | 15/23 |
| **4. Information for mothers about KMC (n=5 facilities)** |  |
| - Availability of brochures and information sheets in the facility | 5/5 |
| - Posters on display | 4/5 |
| - Others like Video | 3/5 |
| **Presence of all domains** | 3/5 |
| **5. Clothing of the baby** |  |
| - Cap | 5/23 |
| - Socks | 3/23 |
| - Nappy | 18/23 |
| - Front open sleeveless shirt | 5/23 |
| **6.. Clothing of the mother/KMC provider** |  |
| - Sari- blouse | 6/18 |
| - Front open- gown | 19/20 |
| **7. KMC Logistics**  **(n=5 facilities)** |  |
| - Beds with back support & pillow | 4/5 |
| - Comfortable arm chairs | 4/5 |
| - Binders to hold infant in KMC position | 5/5 |
| - Curtain for privacy | 3/5 |
| - Diaper | 4/5 |
| - Fan /AC | 5/5 |
| - TV for information/recreation | 4/5 |
| - NG tube | 5/5 |
| - Cribs removed from KMC ward | 4/5 |
| **All of the above** | 2/5 |
| **8. Arrangements important to Quality KMC( n=5 facilities)** |  |
| - Hand washing provision | 5/5 |
| - Toilet facility with water | 4/5 |
| - Waiting space | 0/5 |
| - Maintenance of room temperature | 2/5 |
| **All of the above** | 0/5 |
| **9. Duration of KMC** |  |
| - How long KMC is practiced in the facility |  |
| 1. Continuous | 15/23 |
| 1. Intermittent | 6/23 |
| 1. Whenever possible | 2/23 |
| - Recording of total KMC time/ day in last 24 hours is kept? | 18/23 |
| **10. Position of KMC** |  |
| - Baby placed upright in between mother’s breast | 22/23 |
| - Baby’s head turned to one side, slightly extended | 19/23 |
| - Hips in frog position | 22/23 |
| - Baby’s abdomen at the level of mothers chest | 21/23 |
| - Support from bottom with binder | 21/23 |
| **All of the above** | 18/23 |
| **11. Sleeping and resting position of Mother** |  |
| - Mother maintain the position for KMC (reclined or semi recumbent position) | 20/23 |
| **12. Feeding** |  |
| - Presence of a written feeding policy **(n=5 facilities)** | 4/5 |
| - Nutrition maintenance according to policy (frequency quantity) | 13/23 |
| - Method of feeding practiced by mother |  |
| 1. Exclusive breast feeding | 12/23 |
| 1. Expressed Breast milk with cup-spoon | 6/23 |
| 1. NG tube feeding | 8/23 |
| 1. Bottle feeding | 0/23 |
| **13. Clinical Examination** |  |
| - Temperature is measured 6 hourly 3 consecutive days | 7/23 |
| - Weight gain | 10/23 |
| - Breathing, heart rate, well being | 23/23 |
| - Oxygen requirement | 1/23 |
| - Weekly head circumference measurement (n=17) | 1/17 |
| **14. Discharge Criteria** |  |
| **For Newborn** |  |
| - Nutrition maintained (On breastfeed or Cup/spoon) (n=8) | 8/8 |
| - Target weight gain (consecutive 3 days) (n=8) | 4/8 |
| - Maintenance of temperature (consecutive 3 days) (n=9) | 4/9 |
| - No Oxygen requirement (consecutive 3 days) (n=9) | 4/9 |
| **All of the above** | 1/9 |
| **For mother/care givers** |  |
| - Confident about KMC practice (n=5) | 4/5 |
| - Able to breastfeed or Cup/spoon feed the baby (n=5) | 5/5 |
| - Support of mother from family (n=5) | 4/5 |
| - Mother is well explained about proper KMC practice at home by service provider (n=5) | 1/5 |
| - Mother knows about the danger signs when to return to the facility | 1/5 |
| **All of the above** | 1/5 |
| **15. Routine Follow Up** |  |
| The CHWs follow up the KMC baby at Community Level (n=18) | 1/18 |
| The mother /care giver come to the facility for follow up (n=6) | 1/6 |

**Annex 3 (Tools)**

**In Depth Interview (IDI) Guideline for Care Givers**

1. Please share your experience of providing KMC to your baby at the health facility.

**Probe:**

- What do you know about KMC? (Previous knowledge about KMC; since when, from where learnt about KMC)
- Who explained to you about KMC at the health facility? What did the person say to you about KMC? Did you ask any questions?
- Did you understand the steps of what needs to be done in KMC? Tell us why and how KMC is done.
- Advantage and disadvantage of KMC.
- Effectiveness of KMC

1. What are the advantages and challenges you face while providing KMC to you newborn?

**Probe:**

- Privacy for KMC
- Support (emotional, care of newborn, financial) from health care workers and family members in providing KMC?
- Facilitating factors while practicing KMC at the health facility
- Challenges you face while practicing KMC at the health facility and at home
- Out of pocket expenditure
- Did the health care worker at the facility inform you about continuing KMC after discharge? (Yes/No)

1. What is your opinion about KMC?
2. What is your experience about the process and duration of KMC?

**Probe:**

- Do you think, you can continue to practice KMC at home if needed? If yes, why, if not, why not?
- What kind of help do mothers need in order to practice KMC?

**Key Informant Interview (KII) Guideline for Facility Managers**

1. What is your current position and responsibilities? Since when are you working in the field of maternal, neonatal and child health?
2. What is you experience of implementation of KMC program at your facility?
3. **Challenges and recommendations for implementation and scale-up of KMC at different levels of health facilities**
4. ***Fidelity of adherence of the national KMC guideline***

a) Are newborns weighed at birth in your facility?

b) How can monitoring of KMC by the health facility staff be ensured according to national protocol?

e) How can follow up of KMC of newborns discharged from facility be ensured per protocol?

f) What are the challenges and enabling factors in KMC guideline implementation and how can the challenges be overcome?

g) How can KMC compliance among the care providers be ensured/increased?

1. ***Health system enablers and barriers for implementation of KMC***

**a) Leadership and Governance:**

• Is there a specified quality assurance mechanism and measurement framework for KMC services available at this facility?

• Suggest some measures to increase KMC awareness and commitment among facility managers

**b) Health Service Delivery:**

• Tell us about the quality of KMC services in your facility

• Is there a dedicated space for KMC services available in the health facility?

• Is follow up of KMC after discharge done on regular basis and how do you ensure it?

**c) Health Information System:**

• Are facility based KMC data included at the national HMIS? How to ensure it?

• Is record keeping of KMC and LBW in the health facilities available?

• If yes, whether quality assurance measures are taken to validate available data on KMC and LBW in the health facilities?

• What are the challenges of HMIS in reporting KMC data? How to overcome them?

**d) Health Care Financing:**

• Are adequate funds allocated for KMC in annual budget?

• What role do development partner’s play in KMC implementation financing? Do you think this support is adding value to the KMC programme? If yes, how? If no, why?

• How much is the out-of- pocket expenditure of the care seekers in KMC implementation? Can it be avoided/minimized?

**e) Health Workforce:**

• Number of trained health workers at different levels in implementation of KMC at this facility? Can the number be increased? What can be done to increase it?

- What can be done to ensure quality and retention of training on KMC to the health care workers?

• What is the status of training and refresher training of health workers on KMC? Are refresher trainings available?

• Is mentorship and supervision mechanisms for KMC available/in place?

• What is the Knowledge of the HRH on KMC?

• What is the health worker perception towards KMC?

• Is there an existent job description for health workers supporting KMC?

**f) Essential medical products & technologies:**

• Is there regular provision of adequate resources and supplies needed to perform KMC?

**Key Informant Interview (KII) Guideline for Health Care Providers (Doctors, Nurses)**

1. What is current position and responsibilities, and since when you are working in the Kangaroo Mother Care (KMC) unit?
2. Please share us your experience of working in/ about the KMC unit in your Hospital.

**Probe:**

- Training received on KMC – Last time of training, from where, duration, provision of refresher training; if yes, then how often?
- Knowledge of KMC- need of KMC, benefits of KMC, disadvantage of KMC
- Have you read the KMC guideline?
- Is KMC feasible/ suitable to practice in your facility?
- What is your opinion about KMC implementation in your health facility?
- Do you think it is effective? Why is KMC effective or ineffective in your work environment/ institutional settings?
- What role do other staffs play in KMC implementation? Who is designated for which task?
- Please share us some experiences of some neonates underwent KMC.
- When and how do teach the parents/ caregivers about KMC?
- What do you say/advice during discharge and follow-up of KMC baby?

1. Is it feasible to implement KMC according to guideline? Why/ why not?
2. What are challenges you face normally while working in KMC unit?

**Probe:**

- What are the challenges you face while counseling the mothers to practice KMC?
- Workload
- Dedicated space for KMC
- Follow up of KMC after discharge
- Provision of resources and supplies needed to perform KMC
- Supervision structure/mechanism
- How do you know mothers do practice KMC properly?

1. Please mention few good practices in KMC implementation.
2. What are scopes for improvement?

**KMC observation checklist**

| **BRAC James P Grant School of Public Health, BRAC University** | | |
| --- | --- | --- |
| **Study Topic:** Implementation Research on introducing Kangaroo Mother Care (KMC) services in health facilities in Bangladesh | | |
| General information | | |
| **Theme** | **Response** | **Code** |
| Name of the facility: |  | Ginf1 |
| ID of the facility |  | Ginf2 |
| Name of the observer |  | Ginf3 |
| ID of the Observer |  | Ginf4 |
| Observation Start Date | \|___\|___\|/\|___\|___\|/201\|____\|  Day Month Year | Ginf5 |
| Observation End Date | \|___\|___\|/\|___\|___\|/201\|____\|  Day Month Year | Ginf6 |
| Daily Observation duration | └─┴─┘:└─┴─┘  Hour Minute | Ginf7 |
| Consent taken | 0= No 1= Yes | Ginf 8 |
| Name of the observed mother/care giver: |  | Ginf 9 |
| ID of the observed mother/care giver |  | Ginf 10 |
| Age of the observed mother/care giver: | years | Ginf 11 |
| Name of the Baby |  | Ginf 12 |
| ID of the baby |  | Ginf 13 |
| Age of the baby | Days | Ginf 14 |
| Weight of the baby | ----------------grams | Ginf 15 |

# **Observation Checklist (Q.1-15 will be filled up on 1st day of observation Q. 16-19 will be filled up on consecutive days)**

| **Theme** | **Response** | **Code** |
| --- | --- | --- |
| **Recordkeeping/Documentation available on KMC practice** |  |  |
| Presence and following of - |  |  |
| 1.1 KMC national guideline | 0= No1= Yes | Record1 |
| 1.2 Written feeding policy | 0= No1= Yes | Record2 |
| 1.3 KMC Register | 0= No1= Yes | Record3 |
| 1.4 Daily follow up chart for KMC baby(To be filled in by Nurse) | 0= No1= Yes | Record4 |
| 1.5 KMC Record Form(To be filled in by Doctor) | 0= No1= Yes | Record5 |
| 1.6 KMC services monthly progress report(To be filled in by unit/ward/….in Charge) | 0= No1= Yes | Record6 |
| 1.7 KMC booklet and follow up card | 0= No1= Yes | Record7 |
| **Observation note:** | | |
| **2. Weight:** |  |  |
| 2.1 Was the baby weighted at birth**?** | 0= No1= Yes | Weight1 |
| 2.2. What was weight of the baby at birth (in gram) | -----------------Grams | Weight 2 |
| 2.3 Did the baby fall under the weight for initiating KMC? | 0= No1= Yes | Weight 3 |
| 2.4. Was functioning weighing machine present in the ward? | 0= No1= Yes | Weight 4 |
| **Observation note:** | | |
| **3. Inclusion criteria: Baby**3.1. Under which weight category the baby was included while initiating KMC? | <1200grams=01200-1800grams=1>1800grams=2 | IiniNcbINCaby1nIncmom1cbaby1 Incbaby1 |
| **Observation note:** | | |
| **4. Inclusion Criteria: Mother/KMC provider** |  |  |
| 4.1. Was the Mother /KMC provider willing to provide KMC? | 0= No1= Yes | Incmom1 |
| 4.2. Was Mother /KMC provider fulltime available? | 0= No1= Yes | Incmom2 |
| 4.3 Were other family members available to support mother/KMC provider? | 0= No1= Yes | Incmom3 |
| **Observation note:** | | |
| **5. Exclusion criteria: Baby** Had the baby suffering from any of these conditions while assessing?{Self-reported} |  |  |
| 5.1 Unstable cardiac condition | 0= No1= Yes | Exbaby1 |
| 5.2 With or after major surgery of the newborn | 0= No1= Yes | Exbaby2 |
| 5.3 Unstable respiratory condition | 0= No1= Yes | Exbaby3 |
| 5.4 Any other severe problem specially gross congenital anomalies | 0= No1= Yes | Exbaby4 |
| 5.5 Babies who don’t fit with the stable pre-term criteria | 0= No1= Yes | Exbaby5 |
| **Observation note:** | | |
| **6.Counselling:** |  |  |
| 6.1. Was the mother/Father/Family Member counselled for KMC? | 0= No1= Yes | Coun1 |
| 6.2. Who did the counselling? | Doctor=1  Nurse=2  Midwife=3  Others=4  Please specify--------- | Coun2 |
| 6.3. Presence& use of, Behavior Change Communication material, KMC flip chart &Counselling board during counselling? | 0= No1= Yes | Coun3 |
| **Observation note:** | | |
| **7. Information for mothers about KMC** |  |  |
| 7.1. Availability of brochures and information sheets in the facility | 0= No1= Yes | Inf 1 |
| 7.2. Posters on display | 0= No1= Yes | Inf 2 |
| 7.3. Others like Video | 0= No1= Yes | Inf 3 |
| **Observation note:** | | |
| **8 .Clothing of the baby:** |  |  |
| 8.1 cap | 0= No1= Yes | Clbaby1 |
| 8.2 Socks | 0= No1= Yes | Clbaby2 |
| 8.3 Nappy, | 0= No1= Yes | Clbaby3 |
| 8.4 Front open sleeveless shirt | 0= No1= Yes | Clbaby4 |
| **Observation note:** | | |
| **9 .Clothing of the mother/KMC provider** |  |  |
| 9.1 Sari- blouse | 0= No1= Yes | Clmom1 |
| 9.2 Front open- gown | 0= No1= Yes | Clmom2 |
| **Observation note:** | | |
| **10. KMC Logistics-Presence of** |  |  |
| 10.1 Beds with back support & pillow | 0= No1= Yes | Log1 |
| 10.2 Comfortable arm chairs | 0= No1= Yes | Log2 |
| 10.3 Binders to hold infant in KMC position | 0= No1= Yes | Log3 |
| 10.4 Curtain for privacy | 0= No1= Yes | Log4 |
| 10.5 Diaper | 0= No1= Yes | Log5 |
| 10.6 Fan /AC | 0= No1= Yes | Log6 |
| 10.7 TV for information/recreation | 0= No1= Yes | Log7 |
| 10.8 NG tube | 0= No1= Yes | Log8 |
| 10.9 cribs removed from KMC ward | 0= No1= Yes | Log9 |
| **Observation note:** | | |
| **11. Arrangements important to Quality KMC-**Presence of |  |  |
| 11.1 Hand washing provision | 0= No1= Yes | Qual1 |
| 11.2 Toilet facility with water | 0= No1= Yes | Qual2 |
| 11.3 Waiting space | 0= No1= Yes | Qual3 |
| 11.4Maintenance of room temperature | 0= No 1= Yes | Qual4 |
| **Observation note:** | | |
| **12.Duration of KMC**  12.1. How long KMC is practiced in the facility | Continuous=0 Intermittent=1  Whenever possible=2 | Dur1 |
| 12.2.Recording of total KMC time/ day in last 24 hours is kept? | 0= No1= Yes | Dur2 |
| **Observation note:** | | |
| **13. Position OF KMC-**Did the mother/care giver maintain the position appropriate for KMC |  |  |
| 13.1 Baby placed upright in between mother’s breast | 0= No1= Yes | Pos1 |
| 13.2 Baby’s head turned to one side, slightly extended | 0= No1= Yes | Pos2 |
| 13.3 Hips in frog position | 0= No1= Yes | Pos3 |
| 13.4 Baby’s abdomen at the level of mothers chest | 0= No1= Yes | Pos4 |
| 13.5 Support from bottom with binder | 0= No1= Yes | Pos5 |
| **Observation note:** | | |
| **14. Sleeping and resting position of Mother**14.1. Did the mother maintain the position for KMC (reclined or semi recumbent position) | 0= No1= Yes | Slp1 |
| **Observation note:** | | |
| **15 Feeding** |  |  |
| 15.1. Presence of a written feeding policy | 0= No1= Yes | Feed1 |
| 15.2. Nutrition maintenance according to policy(frequency quantity) | 0= No1= Yes | Feed2 |
| 15.3. Method of feeding practiced by mother | Exclusive breast feeding=0 Expressed Breast milk with cup-spoon=1  NG tube feeding=2  Bottle feeding=3 | Feed3 |
| **Observation note:** | | |
| **16. Clinical Examination:** Whether the daily follow up of KMC baby done according to checklist- |  |  |
| 16.1 Temperature 6hourly 3 consecutive days |  |  |
| Day1 | 0= No1= Yes | Cli 1 |
| Day2 | 0= No1= Yes | Cli 2 |
| Day3 | 0= No1= Yes | Cli 3 |
| 16.2 Weight gain |  |  |
| Day1 | 0= No1= Yes | Cli 4 |
| Day 2 | 0= No1= Yes | Cli5 |
| Day 3 | 0= No1= Yes | Cli6 |
| 16.3 Breathing, heart rate, well being | 0= No1= Yes | Cli7 |
| 16.4 Oxygen requirement |  |  |
| Day1 | 0= No1= Yes | Cli8 |
| Day 2 | 0= No1= Yes | Cli9 |
| Day3 | 0= No1= Yes | Cli10 |
| 16.5 How many hours practiced everyday | Day1…………….hours | Dur3 |
|  | Day2…………….hours | Dur4 |
|  | Day3…………….hours | Dur5 |
| 16.4 Weekly head circumference measurement | 0= No1= Yes | Cli11 |
| **Observation note:** | | |
| **1 7 Discharge Criteria: For Newborn** |  |  |
| 17.1. Nutrition maintained (On breastfeed or Cup/spoon) | 0= No1= Yes | Disbaby1 |
| 17.2. Target weight gain(consecutive 3 days) | 0= No1= Yes | Disbaby2 |
| 17.3 What was the weight of the baby at discharge? | ………….kg | Disbaby3 |
| 17.4. Maintenance of temperature (consecutive 3 days) | 0= No1= Yes | Disbaby4 |
| 17.5. No Oxygen requirement (consecutive 3 days) | 0= No1= Yes | Disbaby5 |
| **Observation note:** | | |
| **18. Discharge Criteria: For mother/care givers** |  |  |
| 18.1. Confident about KMC practice | 0= No 1= Yes | Dismom1 |
| 18.2. Able to breastfeed or Cup/spoon feed the baby | 0= No 1= Yes | Dismom2 |
| 18.3. Support of mother from family | 0= No 1= Yes | Dismom3 |
| 18.4. Mother is well explained about proper KMC practice at home by service provider? | 0= No 1= Yes | Dismom4 |
| 18.5. Mother knows about the danger signs when to return to the facility | 0= No 1= Yes | Dismom5 |
| 18.5 .1 Stops feeding, not feeding well or vomits every thing | 0= No1= Yes | Dismom6 |
| 18.5.2 Restless and irritable, lethargic or unconscious | 0= No1= Yes | Dismom7 |
| 18.5.3 has fever (Temperature above 37.5 degree C | 0= No1= Yes | Dismom8 |
| 18.5.4 Cold ( Temparature below 36.5 Degree C despite rewarmoing for 1 hour) | 0= No1= Yes | Dismom9 |
| 18.5.5 Has convulsions | 0= No1= Yes | Dismom10 |
| 18.5.6 Has difficulty breathing | 0= No1= Yes | Dismom11 |
| 18.5.7 Significant jaundice upto sole and palm | 0= No1= Yes | Dismom12 |
| 18.5.8 Shows any other worrying sign | 0= No1= Yes | Dismom13 |
| **Observation note:** | | |
| **19.Routine Follow Up** |  |  |
| 19.1 Do the CHWs follow up the KMC baby at Community Level | 0= No 1= Yes | Fu1 |
| 19.2. Does the mother /care giver come to the facility for follow up | 0= No 1= Yes | Fu2 |
| **Observation note:** | | |
